# Targeting HER2 Expressing Tumors with a Potent Drug Conjugate Based on an Albumin Binding Domain-Derived Affinity Protein

**DOI:** 10.3390/pharmaceutics13111847

**Published:** 2021-11-03

**Authors:** Javad Garousi, Haozhong Ding, Emma von Witting, Tianqi Xu, Anzhelika Vorobyeva, Maryam Oroujeni, Anna Orlova, Sophia Hober, Torbjörn Gräslund, Vladimir Tolmachev

**Affiliations:** 1 Department of Protein Science, KTH—Royal Institute of Technology, SE-106 91 Stockholm, Sweden; garousi@kth.se (J.G.); haozhong@kth.se (H.D.); emmafog@kth.se (E.v.W.); sophia@kth.se (S.H.); 2 Department of Immunology, Genetics and Pathology, Uppsala University, SE-751 85 Uppsala, Sweden; tianqi.xu@igp.uu.se (T.X.); anzhelika.vorobyeva@igp.uu.se (A.V.); maryam.oroujeni@igp.uu.se (M.O.); vladimir.tolmachev@igp.uu.se (V.T.); 3 Research Centrum for Oncotheranostics, Research School of Chemistry and Applied Biomedical Sciences, Research Tomsk Polytechnic University, RU-634 050 Tomsk, Russia; anna.orlova@ilk.uu.se; 4 Department of Medicinal Chemistry, Uppsala University, SE-751 23 Uppsala, Sweden

**Keywords:** ADAPT, human epidermal growth factor receptor 2, HER2, DM1, albumin binding domain

## Abstract

Albumin binding domain derived affinity proteins (ADAPTs) are a class of small and folded engineered scaffold proteins that holds great promise for targeting cancer tumors. Here, we have extended the in vivo half-life of an ADAPT, targeting the human epidermal growth factor receptor 2 (HER2) by fusion with an albumin binding domain (ABD), and armed it with the highly cytotoxic payload mertansine (DM1) for an investigation of its properties in vitro and in vivo. The resulting drug conjugate, ADAPT6-ABD-mcDM1, retained binding to its intended targets, namely HER2 and serum albumins. Further, it was able to specifically bind to cells with high HER2 expression, get internalized, and showed potent toxicity, with IC_50_ values ranging from 5 to 80 nM. Conversely, no toxic effect was found for cells with low HER2 expression. In vivo, ADAPT6-ABD-mcDM1, radiolabeled with ^99m^Tc, was characterized by low uptake in most normal organs, and the main excretion route was shown to be through the kidneys. The tumor uptake was 5.5% ID/g after 24 h, which was higher than the uptake in all normal organs at this time point except for the kidneys. The uptake in the tumors was blockable by pre-injection of an excess of the monoclonal antibody trastuzumab (having an overlapping epitope on the HER2 receptor). In conclusion, half-life extended drug conjugates based on the ADAPT platform of affinity proteins holds promise for further development towards targeted cancer therapy.

## 1. Introduction

In recent years, delivery of payloads such as toxins, radionuclides, and cytotoxic drugs to cancer cells by affinity proteins have proven to be an efficient route for targeted cancer therapy. Such constructs have become promising agents for the treatment of disseminated cancers [1]. One of the well-studied receptors for targeted cancer therapy is the human epidermal growth factor receptor 2 (HER2), which belongs to the tyrosine kinase receptor family. It is overexpressed in a number of breast, ovarian, lung, and gastrointestinal cancers [2,3]. The differential high-level overexpression on cancer cells and internalizing character has made HER2 one of the particularly suitable receptors to target for delivery of cytotoxic drugs [4].

Drug conjugates typically consist of a protein-based carrier with affinity for a particular molecular abnormality of a malignant cell, and a cytotoxic drug, which is connected by a linker. The most explored type of drug conjugates are antibody drug conjugates (ADCs), where the drug-carrier is a target-specific monoclonal antibody (mAb). ADCs combine several therapeutic modes of action, targeted therapy by the cytotoxic drug, sometimes direct blocking of the normal function of its target, and immunotherapy by the mAb, which invokes immunological reactions (often including an ADCC response). Over the last decade, ADCs have emerged as highly potent anti-cancer drugs for clinical use [5].

Mertansine (DM1) is a commonly used cytotoxic drug that induces cell death by inhibiting polymerization of tubulin and is thus highly effective against rapidly proliferating cells. This is a desirable property of DM1 as it minimizes damage to normal cells with a slower rate of proliferation. Trastuzumab emtansine (T-DM1) is an ADC consisting of DM1 linked to the anti-HER2 mAb trastuzumab. T-DM1 was approved by the US Food and Drug Administration (FDA) in 2013 for the treatment of HER2 positive breast cancer. Patients with acquired resistance to trastuzumab or taxane therapy have been shown to benefit from T-DM1 treatment [6].

Despite the benefits of ADCs in terms of safe delivery of the cytotoxic drug, there are sub-optimal properties of many ADCs in pre-clinical and clinical development, which limits their efficiency. These include a variability of the drug to antibody ratio (DAR) due to random conjugation, inefficient penetration of solid tumors due to the rather large size of the mAb, and possible alteration of the binding properties of the mAb due to random conjugation (potentially to sites involved in antigen binding) [7]. Studies concerning site-specific attachment of drugs to mAbs have been published in recent years [8], but these methodologies are not yet commonly used in FDA approved ADCs.

The use of small engineered scaffold proteins (ESPs) as targeting agents instead of mAbs might be a viable route to overcome some of the inherent problems of random drug attachment. For example, if the ESP does not contain cysteines in the framework, it is possible to introduce one or more cysteines at desired positions in the molecule for site-specific conjugation of the drug. Also, the small size of most ESPs should provide a better penetration of solid tumors and consequently enhance the drug conjugate’s therapeutic effect [9,10,11]. In the case of HER2, clinical imaging studies have shown that different classes of ESPs (e.g., affibody molecules, ADAPTs, DARPins) specifically and efficiently accumulate in tumors expressing the receptor, which suggest that they can potentially be suitable carriers of cytotoxic drugs for cancer therapy [6,12,13,14].

Albumin binding domain (ABD)-derived affinity proteins (ADAPTs) constitute a class of ESPs which have been used for radionuclide targeting applications [15]. ADAPTs are small (5 kDa), folded domains, derived from the G148-GA3 albumin binding domain of streptococcal protein G [16,17,18]. From combinatorial libraries where surface amino acids have been randomized, binders against desired targets can be generated [16]. ADAPT6 is a specific binder to HER2 with an equilibrium dissociation constant of 2 nM [17]. We have previously evaluated different aspects of molecular design of ADAPT6 for radionuclide molecular imaging and have found that ADAPT6 preserves binding to the HER2 target, even after chemical modification such as conjugation with different chelators [19,20,21,22,23,24]. Moreover, a clinical study demonstrated that injections of ^99m^Tc-labelled ADAPT6 were safe, and that ADAPT6 accumulated in HER2-expressing breast cancer with high specificity [14].

Previously, we have found that it is possible to extend the plasma half-life of ESPs by fusion to an albumin binding domain (ABD), which consequently should improve its bioavailability [25,26,27,28]. Half-life extension occurs by binding of the ABD to serum albumin in the blood, leading to an increase in the overall size of the complex that exceeds the filtration cut-off of the kidneys (approximately 60 kDa). A particularly useful ABD variant is ABD_035_ with femtomolar binding affinity (K_D_) to human serum albumin (HSA) [26]. Moreover, we have demonstrated that an ABD-fused ADAPT6 labeled with ^177^Lu can be used to efficiently target human cancer xenografts with high HER2 expression in mice [28]. It was also found that the relative position of ABD and ADAPT6 influences the biodistribution, and that placement of the ABD at the C-terminus of ADAPT6 was particularly favorable [28]. We have therefore used an analogous architecture for the drug conjugates in this study, with ADAPT6 followed by the ABD.

A high hepatic uptake of cytotoxic drug conjugates might cause severe drug induced liver injury (DILI), and hepatic uptake should therefore be minimized [29]. Lipophilic patches in proteins are a main driver of hepatic uptake. Previous studies by our groups concerning ABD-fused affibody molecules conjugated with mcDM1 suggested that the use of multiple hydrophilic amino acids as a spacer between the protein part and mcDM1 reduced the lipophilicity of the drug conjugates, and consequently liver uptake, while tumor uptake and potency remained intact [10,11,30].

Given the excellent properties of radiolabeled ADAPTs as probes for molecular imaging, we have in this study sought to investigate their properties to carry cytotoxic drugs to tumor cells. The HER2-targeting ADAPT6 was expressed as a fusion protein with ABD_035_. A C-terminal cysteine residue was incorporated into the construct, which was used to conjugate DM1 via a non-cleavable maleimidocaproyl (mc) linker, resulting in the drug conjugate ADAPT6-ABD-mcDM1. A non-toxic control protein was also created, ADAPT6-ABD-AA, where the C-terminal cysteine residue was capped with iodoacetamide. A non-targeted control drug conjugate was created, ADAPT_Neg_-ABD-mcDM1, which included ADAPT_Neg_, not interacting with any target, fused to the ABD and derivatized with mcDM1 on the C-terminal cysteine residue. In all variants, a tag was placed at the N-terminus, with the amino acid sequence His-Glu-His-Glu-His-Glu to be used as a chelator for labelling with ^99m^Tc(CO)_3_. The linker connecting ADAPT6 and the ABD had the amino acid sequence (Ser-Ser-Ser-Gly)_3_. Since our previous studies on affibody molecules have shown that the use of multiple hydrophilic amino acids as a spacer between the protein part and mcDM1 reduced liver uptake, we introduced a Glu-Glu-Glu spacer between the ABD and the C-terminal cysteine onto which mcDM1 was conjugated. The biochemical properties and cytotoxic potential of the ADAPT-based drug conjugates was investigated. The biodistribution was further evaluated in nude mice bearing HER2-overexpressing SKOV-3 tumors.

## 2. Materials and Methods

### 2.1. General

All chemicals were obtained from Sigma-Aldrich (St. Louis, MO, USA) or Merck (Darmstadt, Germany). Restriction enzymes were purchased from New England Biolabs (Ipswitch, MA, USA). Radioactivity was measured by an automated γ-spectrometer with a NaI(Tl) detector (1480 Wizard; Wallac Oy, Turku, Finland).

### 2.2. Production and Purification of ADAPT Fusion Proteins

Genes encoding the HER2-binding ADAPT6-ABD and the non-targeted ADAPT_Neg_-ABD fusion proteins were synthesized by Thermo Fisher Scientific (Waltham, MA, USA). The genes were PCR amplified, adding nucleotides encoding the N-terminal amino acid sequence MHEHEHEDANS and the C-terminal amino acid sequence EEEC as well as sites recognized by the restriction enzymes NcoI and AscI. The PCR-products were sub-cloned into the expression vector pET-21a(+) by restriction with NcoI and AscI followed by ligation. The proteins were expressed in *Escherichia coli* BL21 (DE3) star cells at 25 °C for 24 h after induction by isopropyl β-D-1-thiogalactopyranoside to a final concentration of 1 mM. The intracellular fraction was released by sonication. The proteins were purified by affinity chromatography on a column with immobilized human serum albumin (HSA) as previously described [28]. Briefly, the cell lysates were loaded on the column after equilibration with 1xTST buffer (25 mM Tris-HCl, 1 mM EDTA, 0.2 M NaCl, 0.05% Tween 20, pH 8.0), followed by washing with 1xTST and 5 mM NH_4_Ac (pH 5.5). Bound proteins were eluted with 0.5 M acetic acid (pH 2.8) and were lyophilized.

### 2.3. Conjugation with mcDM1

The ADAPT fusion proteins were conjugated with mcDM1 (Levena Biopharma, San Diego, CA, USA) at a molar ratio of 3:1 (drug:protein) through coupling of the maleimide group in the mc-linker with the free thiol group of the C-terminal cysteine in the fusion proteins. Before conjugation, the lyophilized fusion proteins were dissolved in 10 mM Tris-HCl buffer (pH 7.85). Potentially oxidized thiol groups were reduced by tris (2-carboxyethyl) phosphine (TCEP) at a final concentration of 5 mM for 30 min at 37 °C. The pH was adjusted to 6.5 using HCl. The fusion proteins (5 mg/mL) were mixed with mcDM1 and were incubated at 25 °C for 1 h. The non-toxic control ADAPT6-ABD-AA was produced by alkylation of the C-terminal cysteine with 2-iodoacetamide. ADAPT6-ABD was dissolved in alkylation buffer (0.2 M NH_4_HCO_3_, pH 8.0) and was incubated with TCEP as described above, to reduce potentially oxidized cysteines. 2-iodoacetamide was added to a final concentration of 10 mM followed by incubation at room temperature for 30 min in the dark. Both ADAPT drug conjugates (ADAPT-DCs) and the non-toxic control were purified through RP-HPLC on an Agilent 1260 series Infinity II machine (Agilent, Santa Clara, CA, USA). The reaction mixtures after conjugation were diluted 1:1 with buffer A (0.1% trifluoroacetic acid in water) and was loaded on a Zorbax 300SB-C18 semi-preparative column (Agilent). Bound constructs were eluted using a gradient of 30–70% of buffer B (0.1% trifluoroacetic acid in acetonitrile) over 30 min using a flow rate of 3 mL/min. The fractions containing the constructs of interest were pooled, lyophilized and were stored at −80 °C until use.

### 2.4. Biochemical Characterization

The identity of ADAPT-DCs and the non-toxic control were analyzed by SDS-PAGE (Biorad, Hercules, CA, USA) under reducing conditions. The oligomeric state of the constructs was analyzed by size exclusion chromatography, by passage through a 5/150 column (GE Healthcare, Uppsala, Sweden), packed with Superdex-75, using a flow rate of 0.45 mL/min in PBS. The molecular weight of purified ADAPT variants was measured by liquid chromatography-electro-spray ionization-mass spectrometry (LC-ESI-MS) using an Impact II UHR QqTOF MS (Bruker Daltonics, Billerica, MA, USA). The purity of the constructs was evaluated by RP-HPLC using a Zorbax 300SB-C18 column (Agilent) using a gradient from 30–60% of buffer B over 20 min at a flow rate of 1 mL/min.

### 2.5. Biosensor Analysis on a Biacore Instrument

Target binding analysis was performed on a Biacore 3000 instrument (GE Healthcare) by injecting samples over a CM5 chip. The chip was prepared by immobilizing murine serum albumin (MSA) (Sigma-Aldrich) to a response level of ~500 RU in one channel, human serum albumin (HSA) (Novozymes, Bagsvaerd, Denmark) to ~400 RU in a second channel, and HER2-Fc fusion protein (R&D Systems, Minneapolis, MN, USA) to ~1000 RU in a third channel. A blank surface for normalization was also created in a fourth channel by activation and deactivation. The experiments were preformed essentially as described previously [31]. Briefly, a series of five different concentrations of each analyte in 1xPBST (PBS supplemented with 0.05% Tween 20, pH 7.4) was sequentially injected over the flow-cells. The injections were carried out with a flow rate of 30 μL/min, for 300 s for binding, followed by 1200 s for dissociation of the analytes.

### 2.6. Cell Culture

The cancer cell lines, AU565, SKBR3, SKOV-3, A549, and MCF7 were purchased from American Type Culture Collection (ATCC, via LGC Promochem, Borås, Sweden) and were grown in RPMI-1640 (SKOV-3, SKBR3, AU565), or Dulbecco’s modified Eagle medium (A549, MCF7) (Cytiva Hyclone, Uppsala, Sweden) supplemented with 10% FBS (Sigma-Aldrich) in a humidified incubator at 37 °C in a 5% CO_2_ atmosphere.

### 2.7. In Vitro Cytotoxicity Analysis

AU565, SKBR3 A549, and MCF7 (5000 cells/well) or SKOV-3 (2000 cells/well) were seeded in 96-well plates and cultured overnight. On the next morning, the medium was aspirated and increasing concentrations of ADAPT-DCs or controls were prepared in four replicates, by dilution in a fresh media, and were added to the wells. The plates were incubated for 72 h in a humidified incubator at 37 °C in a 5% CO_2_ atmosphere. Cell viability was determined using a Cell Counting Kit-8 (CCK-8, Sigma-Aldrich) according to the manufacturer’s protocol. The obtained data were analyzed using nonlinear regression in Prism (GraphPad Software, San Diego, CA, USA).

### 2.8. Radiolabeling

^99m^TcO_4^−^_ was obtained by elution from a ^99^Mo/^99m^Tc generator (Mallinckrodt, Petten, The Netherlands) with sterile 0.9% NaCl.

Site-specific radiolabeling of ADAPT-DCs and ADAPT6-ABD-AA with [^99m^Tc(CO)_3_]^+^ was performed as described earlier [21,32,33]. A CRS kit (PSI, Villigen, Switzerland) was used to produce [^99m^Tc(CO)_3_(H_2_O)_3_]^+^ from ^99m^TcO_4_^−^ for labeling of the N-terminal His-Glu-His-Glu-His-Glu sequence in the constructs with ^99m^Tc. The labeled compounds were purified using NAP-5 columns (GE Healthcare). The radiochemical yield and radiochemical purity were determined by radio iTLC using iTLC silica gel strips (Varian, Lake Forest, CA, USA) with subsequent measurement using a Cyclone phosphor system (PerkinElmer, Waltham, MA, USA). Additionally, the radio-iTLC data was cross-validated by high performance liquid chromatography (HPLC) analysis on a Hitachi Chromaster HPLC system with radioactivity detector and a Luna RP C18 column at room temperature. The samples were diluted in buffer A (0.1% trifluoroacetic acid in water) and loaded onto the column. The samples were eluted using a gradient of 5–80% of buffer B (0.1% trifluoroacetic acid in acetonitrile), using a flow rate of 1 mL/min over 20 min.

### 2.9. In Vitro Characterization of the ^99m^Tc-Labeled Constructs

In vitro specificity tests were performed according to a method described by Wållberg et al. [31]. Briefly, 1 × 10^6^ SKOV-3 or AU565 cells were seeded in six-well plates on the day before the experiment. The dishes were divided into two sets. In the binding sets, radiolabeled constructs were added to reach a concentration of 50 nM. In the control sets, HER2 receptors on the cells were saturated with 50 mM of non-radiolabeled proteins 15 min before adding the labeled compounds. After incubation for 1 h at 37 °C, cells were washed, and the cell-associated radioactivity was measured using a γ-spectrometer.

Analysis of cellular processing was performed according to a method described by Wållberg et al. [31]. In brief, 1 × 10^6^ SKOV-3 and AU565 cells were seeded in six-well plates on the day before the experiment. Cells were incubated at 37 °C with 50 nM of radiolabeled constructs. At 1, 2, 4, 8, and 24 h after addition of the constructs, media was aspirated and the membrane-bound fraction was released by acid wash using 4 M urea in 0.2 M glycine buffer, pH 2.0. The internalized fraction was released by incubating with 1 M NaOH. The radioactivity of the fractions was measured by a γ-spectrometer. Prism (version 8.00 for Windows, GraphPad Software) was used to analyze cellular processing using an unpaired two-tailed *t*-test.

The binding affinity of the radiolabeled constructs to HER2-expressing SKOV-3 cells was measured using a LigandTracer biosensor (Ridgeview Instruments, Uppsala, Sweden) based on a method described previously [34].

### 2.10. Biodistribution in Tumor Bearing Mice

Animal studies were planned in agreement with EU Directive 2010/63/EU for animal experiments and Swedish national legislation concerning the protection of laboratory animals and were approved by the Ethics Committee for Animal Research in Uppsala, Sweden (Permit Number: C4/2016).

Biodistribution and targeting properties of the labeled compounds were evaluated in female BALB/c nu/nu mice bearing HER2-positive SKOV-3 xenografts. The xenografts were established by subcutaneously implanting 1 × 10^7^ SKOV-3 cells in a hind leg, three weeks before the experiment.

The mice were randomized into six groups with four mice per group. Each mouse received a tail vein injection of 6 μg of ^99m^Tc-ADAPT6-ABD-mcDM1 in 100 μL PBS containing 1% BSA and the biodistribution was measured 1, 4, 24, and 48 h after injection. The injected activities were 60 kBq/mouse for mice dissected 1 and 4 h after injection, 640 kBq/mouse for mice dissected 24 h after injection, and 10.2 MBq for mice dissected 48 h after injection. After exsanguination under anesthesia (25 μL/g body weight; ketamine 10 mg/mL; Rompun 1 mg/mL), the organs and tissues of interest were excised, weighed, and their activity was measured using an automated γ-spectrometer.

To confirm the specificity of ^99m^Tc-ADAPT_6_-ABD-mcDM1 accumulation in the tumors, two control experiments were performed. One group of four mice was intravenously injected with ^99m^Tc-ADAPT_Neg_-ABD-mcDM1 (6 µg, 640 kBq), and the biodistribution was measured 24 h after injection. Another group of four mice was subcutaneously injected with trastuzumab (8.4 mg/per mouse) to block HER2 receptors. 24 h after injection of trastuzumab, the mice were injected with ^99m^Tc-ADAPT6-ABD-mcDM1 (6 µg, 640 kBq), and the biodistribution was measured an additional 24 h later.

For confirmation of the biodistribution results obtained by the ex vivo measurements, a SPECT/CT imaging was performed. Two mice were injected with ^99m^Tc-ADAPT_6_-ABD-mcDM1 (6 µg, 30 MBq). One mouse was pre-injected 24 h before injection of ^99m^Tc-ADAPT6-ABD-mcDM1 with 8.4 mg trastuzumab. Imaging of the mice was performed 24 h after injection of radiolabeled ADAPT6-ABD-mcDM1 using a nanoScan SPECT/CT scanner (Mediso Medical Imaging Systems, Budapest, Hungary). Immediately before being placed in the camera, the mice were euthanized by CO_2_ asphyxiation. The acquisition time was 60 min. The camera settings were as described previously [35].

## 3. Results

### 3.1. Production, Purification, Conjugation and Biochemical Characterization

**Figure 1:**
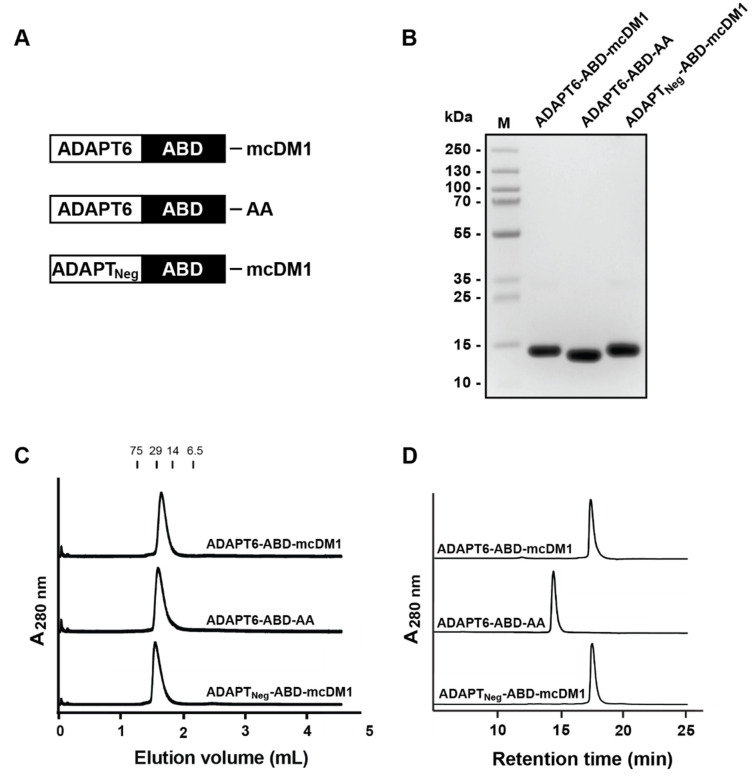
Biochemical characterization. (**A**) Schematic description of the ADAPT drug conjugates (ADAPT-DCs) and the non-toxic control ADAPT6-ABD-AA. A (Ser_3_Gly)_3_-linker was used to connect the ADAPT and ABD domains in all constructs. (**B**) SDS-PAGE analysis under reducing conditions. 5 μg material was loaded in each lane. The molecular weights of marker proteins in lane M are shown on the left side. (**C**) Size-exclusion chromatography analysis. Indicated above the chromatograms are elution volumes of marker proteins. The molecular weights (in kDa) of the marker proteins are also indicated above the chromatograms. (**D**) RP-HPLC analysis using a linear gradient (30–60%) of acetonitrile in water with 0.1% TFA during 20 min.

The two fusion proteins, ADAPT6-ABD and ADAPT_Neg_-ABD, were produced in *Escherichia coli* and were purified, followed by conjugation of mcDM1 to the C-terminal cysteine residue (Figure 1A). A non-toxic control was created, where the C-terminal cysteine of ADAPT6-ABD was alkylated with iodoacetamide (Figure 1A). The purity and molecular weight of the constructs were evaluated by SDS-PAGE analysis under reducing conditions (Figure 1B and Figure S1). The gel confirmed that the proteins were of high purity and with essentially the expected molecular weight. The mono/oligomeric state of the constructs was further investigated by size exclusion chromatography under native conditions. The constructs were eluted as single peaks at the expected elution volumes, confirming no degradation or aggregation (Figure 1C). The proteins were also analyzed by RP-HPLC (Figure 1D). The purity was estimated by calculation of the area-under-curve in the obtained chromatograms, and was found to be more than 95%. ADAPT6-ABD-AA, the non-toxic control, was eluted earlier than the two drug conjugates, showing an increase in hydrophobicity imposed by mcDM1. The molecular weights of the proteins were measured by LC-MS and the obtained results were in good agreement with the theoretical values with a difference less than 1 Da (Table S1).

**Figure 2:**
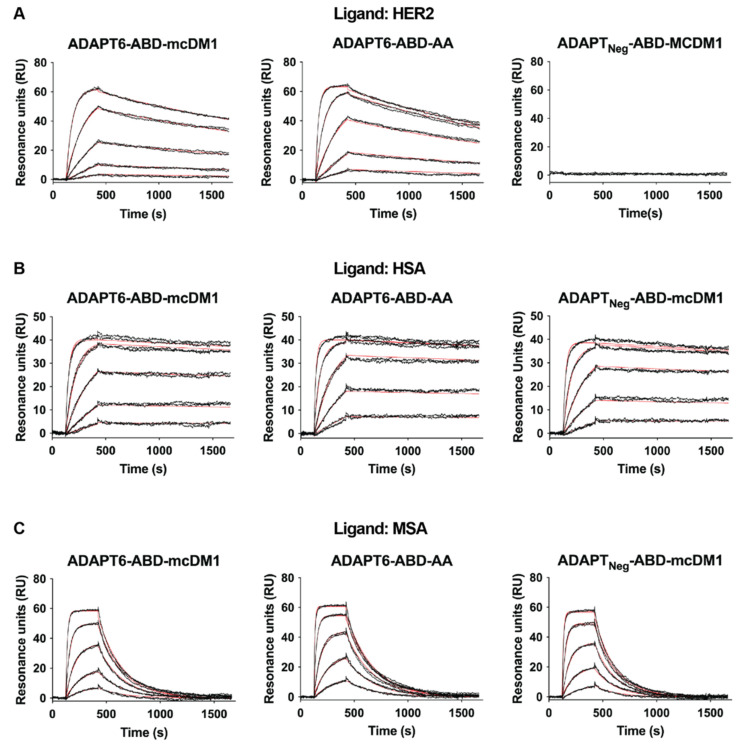
Analysis of the interactions between the constructs and different ligands. The analysis was conducted using a Biacore real-time biosensor instrument. Dilution series of the constructs, indicated above each paned, were injected over the chip surface with different immobilized ligands (**A**) HER2, (**B**) HSA and (**C**) MSA. The panels are overlays of the sensorgrams (in black) obtained from two identical injections for each concentration and the theoretical curves drawn using the calculated kinetic parameters for each interaction (in red).

The affinity of the conjugates to HER2, HSA and MSA were measured using an SPR, real-time biosensor, instrument. The kinetic constants and equilibrium dissociation constants (K_D_ values) were calculated based on the recorded data from the interaction between the immobilized ligands (HER2, HSA or MSA) and different injected concentrations of analytes (Figure 2 and Table 1). The affinities (K_D_) for ADAPT6-ABD-mcDM1 and ADAPT6-ABD-AA interacting with HER2 were 6 and 3 nM, respectively, showing that mcDM1 conjugation did not appreciably affect the affinity of ADAPT6 to HER2. As expected, the non-target control ADAPT_Neg_-ABD-mcDM1 did not show any interaction with HER2. The affinities of the conjugates to HSA were strong and varied between 0.1 and 0.5 nM. The affinities to MSA were weaker and varied between 4 and 20 nM. The weaker affinity to MSA compared to HSA was mainly a consequence of a faster dissociation rate. A comparison of ADAPT6-ABD-mcDM1 and ADAPT6-ABD-AA showed that mcDM1 conjugation lowered the affinity for HSA and MSA by approximately five-fold.

### 3.2. In Vitro Cytotoxicity Analysis

**Figure 3:**
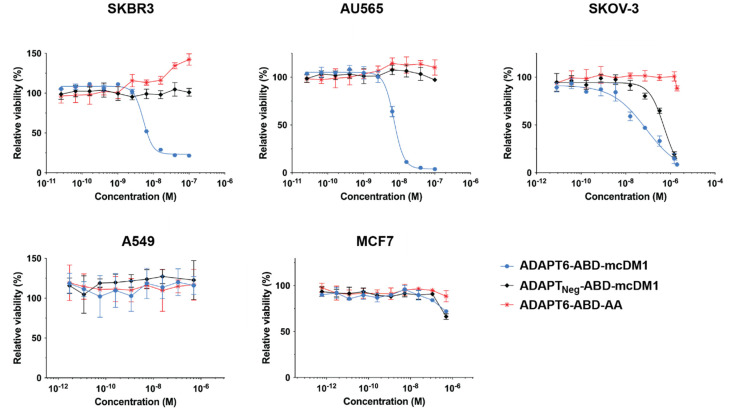
In vitro cytotoxicity of the ADAPT constructs. The cytotoxicity was measured by the treatment of different cell lines, as indicated above the panels, with serial dilutions of the conjugates. The viability at each data point was normalized to the viability of each cell line in complete media without conjugate (which was set to 100%). Each data point is an average of four replicates of each concentration ± 1 SD.

The cytotoxic potential of ADAPT6-ABD-mcDM1 was investigated by incubation of dilution series of the conjugate with cell lines having different HER2-expression levels (Figure S2) and the results are displayed in Figure 3. The non-toxic control, ADAPT6-ABD-AA, and the non-targeted control ADAPT_Neg_-ABD-mcDM1 were also included in this experiment.

For high-HER2 expressing cells (SKBR3, AU565, and SKOV-3), ADAPT6-ABD-mcDM1 demonstrated a dose-dependent cytotoxic effect. The IC_50_ values were 5 nM, 7 nM, and 80 nM for SKBR3, AU565 and SKOV-3, respectively (Figure 3). The non-toxic control, ADAPT6-ABD-AA, did not show any toxic effect on any of the cells with high HER2 expression in the range of concentrations tested, except for a slight stimulation of SKBR3 proliferation at high concentrations. The non-targeted control, ADAPT_Neg_-ABD-mcDM1 did not affect the viability of AU565 or SKBR3. However, it showed a weak effect on the SKOV-3 cell line, with an IC_50_ value of 500 nM, which was six-fold weaker than the IC_50_ value of the targeted ADAPT6-ABD-mcDM1. No effect on viability was observed for A549 or MCF7 cells, both with low HER2, after incubation with ADAPT6-ABD-mcDM1 or the two controls, further suggesting that the cytotoxic action was HER2 mediated and that a relatively high level of HER2 expression is required for the intended effect.

### 3.3. Radiolabeling

The three constructs were site-specifically labeled with ^99m^Tc via the N-terminal His-Glu-His-Glu-His-Glu-tag. The radiochemical yield for all three constructs was over 70%. After purification, the radiochemical purity was over 99% as determined with RP-HPLC (Figure S3). The radiochemical yield and purity data are summarized in Table S2. To investigate the stability of the label, the conjugates were challenged with a 5000-fold molar excess of histidine. No release of free ^99m^Tc was observed after the histidine challenge, showing robust labeling of the constructs (Table S2).

### 3.4. In Vitro Characterization of the Radiolabeled Constructs

**Figure 4:**
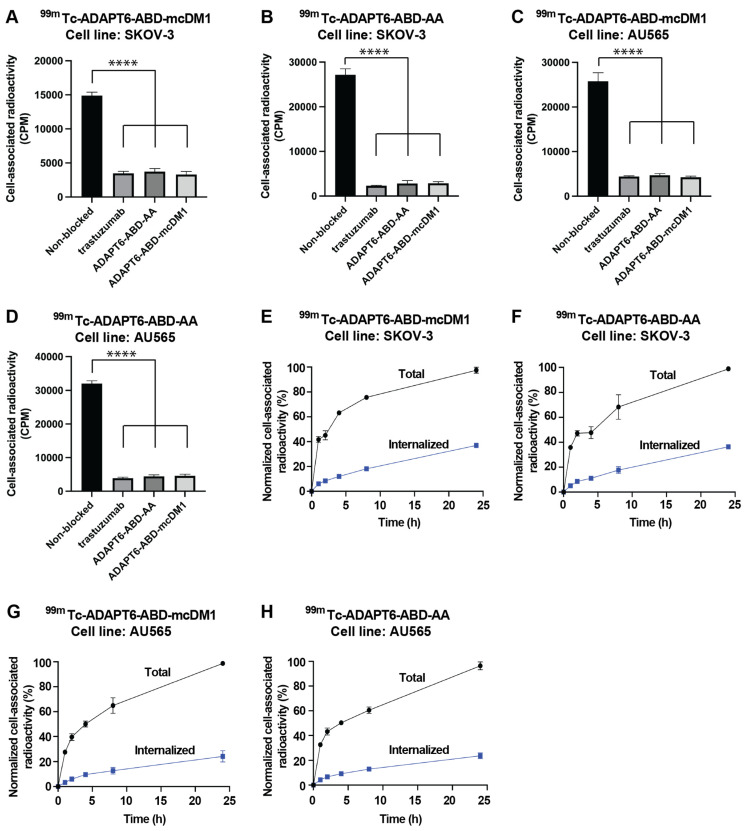
Binding specificity and cellular processing of ^99m^Tc-labeled ADAPT6 constructs. (**A**–**D**) SKOV-3 or AU565 cells were incubated with ^99m^Tc-ADAPT6-ABD-mcDM1 or ^99m^Tc-ADAPT6-ABD-AA, indicated above each panel. For pre-saturation of HER2 receptors, a 100-fold molar excess of non-radiolabeled trastuzumab, ADAPT6-ABD-AA or ADAPT6-ABD-mcDM1 was added to the cells. The data are mean values of radioactivity measured in three cell dishes ± 1 SD. (**E**–**H**) Cellular processing of ADAPT6-ABD-AA and ADAPT6-ABD-mcDM1 by SKOV-3 and AU565 cells during 24 h. The constructs and cell lines are indicated above each panel. The data were normalized to the average of maximum cell associated radioactivity for each radioconjugate, which was set to 100%. The data are mean values of radioactivity measured in three cell dishes ± 1 SD. The significance indicator **** correspond to *p* < 0.0001.

The binding of the ^99m^Tc-labeled constructs to HER2 expressing SKOV-3 and AU565 cells was investigated by a saturation assay. The cells were attached to the bottom of a 96-well plate and the radiolabeled ADAPT6 conjugates were added to the wells with or without pre-saturation of available HER2 receptors on the cells with each ADAPT6 construct or the mAb trastuzumab (Figure 4). The binding of all constructs was significantly (*p* < 0.0001) reduced by pre-saturation of the receptors using trastuzumab or any of the ADAPT6 constructs. The results further corroborate that the binding of the constructs to the cells is HER2 mediated and that they bind to an epitope that overlaps trastuzumab’s epitope.

Next, the rate of cell association, internalization and processing of ^99m^Tc-ADAPT6-ABD-mcDM1 and ^99m^Tc-ADAPT6-ABD-AA were investigated (Figure 4). Binding to SKOV-3 and AU565 cells, with high HER2 expression, was initially fast, but increased more slowly between 2 and 24 h. The internalization of the constructs was relatively slow and increased throughout the experiment. The maximum internalized fraction at 24 h for SKOV-3 cells was 38% and 37% for of ^99m^Tc-ADAPT6-ABD-mcDM1 and ^99m^Tc-ADAPT6-ABD-AA, respectively. The internalized fraction at 24 h for AU565 cells was 20% for both constructs (Figure 4).

**Figure 5:**
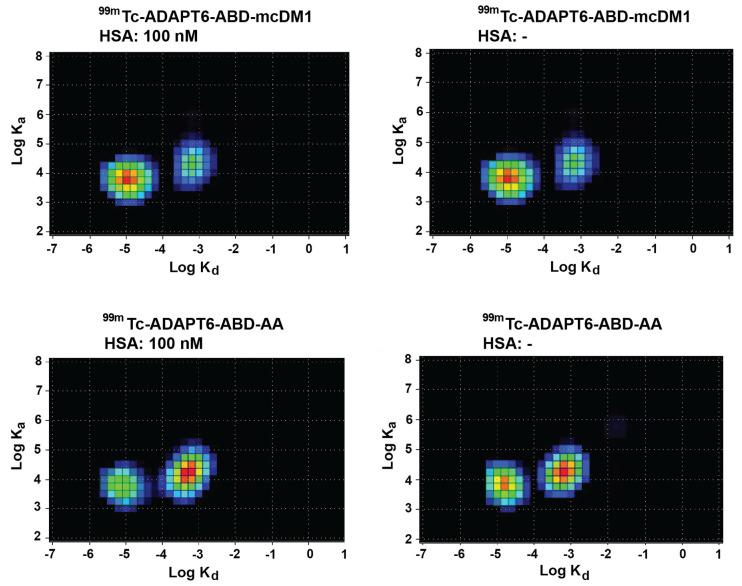
Interaction maps. The interaction of ^99m^Tc-ADAPT6-ABD-mcDM1 and ^99m^Tc-ADAPT6-ABD-AA with SKOV-3 cells was analyzed in a LigandTracer biosensor in the absence or presence of HSA (100 nM), and interaction maps were generated from the recorded data. The warmer colors in the map correspond to a larger degree of contribution to the interaction. The x-axes correspond to the logarithm of the dissociation rate (k_d_) and the y-axes correspond to the logarithm of the association rate (k_a_). The data demonstrate two interactions with similar association rates but with different dissociation rates.

The affinity of ^99m^Tc-ADAPT6-ABD-mcDM1 and ^99m^Tc-ADAPT6-ABD-AA to SKOV-3 cells was measured using a LigandTracer real-time biosensor. The experiments were performed with or without the addition of HSA (100 nM), to more accurately mimic the in vivo milieu and to understand if HSA-complexation affects the ability of the constructs to interact with HER2 expressing cells. Interaction maps of the experiments were generated and are presented in Figure 5. Both ^99m^Tc-ADAPT6-ABD-mcDM1 and ^99m^Tc-ADAPT6-ABD-AA demonstrated two modes of interaction with the cells, a strong and a weak. The strong interaction was centered around 1 nM in all cases and the weaker interaction had affinities that ranged between 25 and 45 nM. There was no difference in the affinities in the presence or absence of HSA, indicating that HSA-complexation did not affect the ability of ^99m^Tc-ADAPT6-ABD-mcDM1 or ^99m^Tc-ADAPT6-ABD-AA to interact with the cells.

### 3.5. Biodistribution in Tumor Bearing Mice

**Figure 6:**
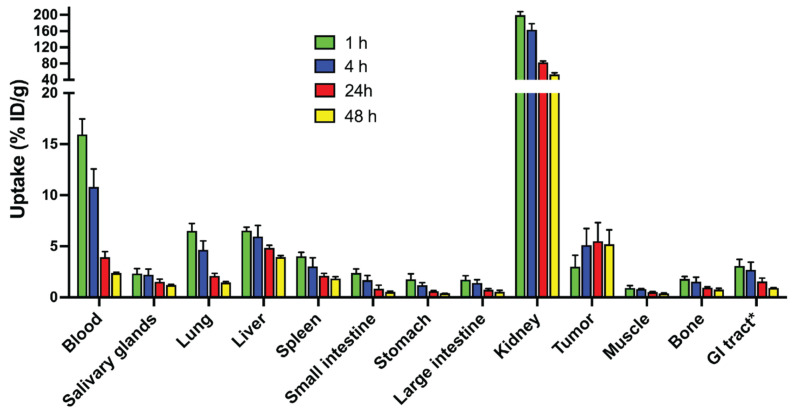
Biodistribution of ^99m^Tc-ADAPT6-ABD-mcDM1 in BALB/c nu/nu mice bearing HER2 expressing SKOV-3 xenografts at 1, 4, 24, and 48 h after injection. The radioactivity was calculated as percent of injected dose per gram tissue (% ID/g) and presented as the mean value from four mice ± 1 SD. * Data for gastrointestinal (GI) tract with content is presented as % ID per whole sample.

The biodistribution of ^99m^Tc-labeled ADAPT6-ABD-mcDM1 was evaluated in BALB/c nu/nu mice bearing HER2 expressing SKOV-3 xenografts at 1, 4, 24, and 48 h after injection (Figure 6, Table S3). At all time-points, the highest uptake was in the kidneys. The radioactivity in blood was over 15% ID/g at 1 h p.i. and over 2% at 48 h p.i. This shows the efficiency of fusion with the ABD for the prolongation of blood retention. The elimination half-life was determined to be 9.0 h. The tumor uptake reached a plateau by 4 h p.i. At 24 h, the average tumor uptake was higher than all other normal organs except the kidneys. Liver uptake was elevated, which is an indication of a hydrophobic character of the conjugate.

**Figure 7:**
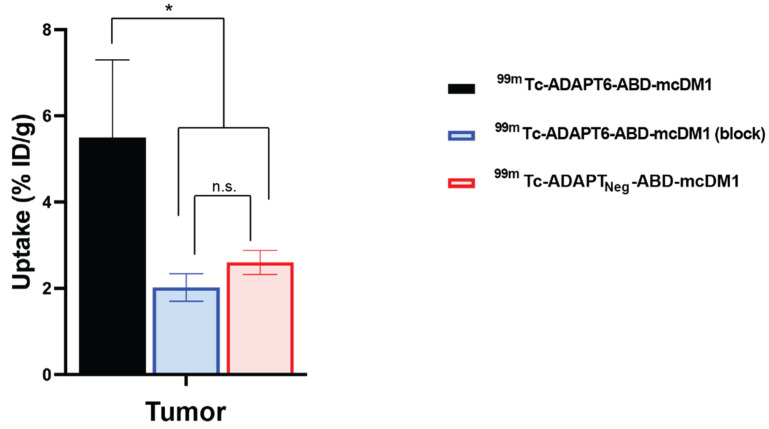
Specificity of targeting of HER2-expressing SKOV-3 xenografts using ^99m^Tc-ADAPT6-ABD-mcDM1. The uptake of radioactivity in the tumors at 24 h after injection of ^99m^Tc-ADAPT6-ABD-mcDM1 with pre-saturation with trastuzumab, ^99m^Tc-ADAPT6-ABD-mcDM1 (block), or without pre-saturation with trastuzumab, ^99m^Tc-ADAPT6-ABD-mcDM1, or after injection of the non-targeting control ^99m^Tc-ADAPT_Neg_-ABD-mcDM1. The radioactivity was calculated as percent of injected dose per gram tumor (% ID/g) and is presented as the mean value from four mice ± 1 SD. The tumor uptake of ^99m^Tc-ADAPT6-ABD-mcDM1 was significantly higher than the uptake of the same construct after blocking or the uptake of ^99m^Tc-ADAPT_Neg_-ABD-mcDM1. The significance indicator * corresponds to *p* < 0.05. n.s. (not significant) corresponds to *p* > 0.05.

By blocking available receptors with trastuzumab or by injecting the non-targeting drug conjugate ^99m^Tc-ADAPT_Neg_-ABD-mcDM1, a specificity test to investigate if tumor uptake was HER2 mediated, was performed. The tumor uptake of ^99m^Tc-ADAPT6-ABD-mcDM1 was significantly (*p* < 0.05) higher before than after trastuzumab blocking (Figure 7). The tumor uptake of ^99m^Tc-ADAPT6-ABD-mcDM1 was also significantly (*p* < 0.05) higher than the uptake of ^99m^Tc-ADAPT_Neg_-ABD-mcDM1. This demonstrates a HER2-dependent uptake of ^99m^Tc-ADAPT6-ABD-mcDM1 in SKOV-3 xenografts.

**Figure 8:**
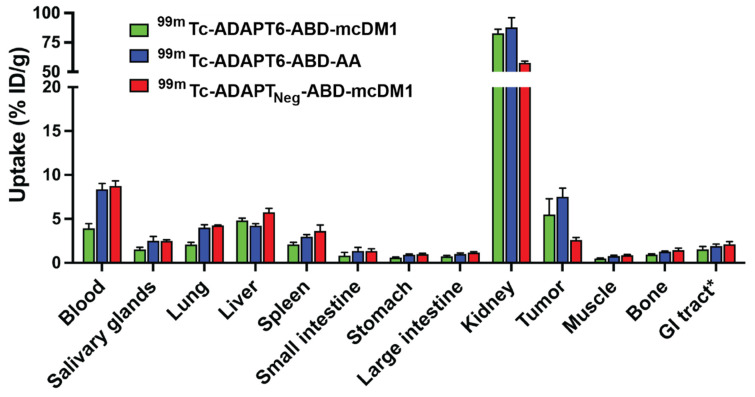
Comparison of biodistribution of ^99m^Tc-ADAPT6-ABD-mcDM1, ^99m^Tc-ADAPT6-ABD-AA and ^99m^Tc-ADAPT_Neg_-ABD-mcDM1 conjugates at 24 h. The radioactivity is calculated as a percent of injected dose per gram tissue (% ID/g) and presented as mean value from four mice ± 1 SD. * Data for gastrointestinal (GI) tract with content is presented as % ID per whole sample.

Further, the biodistribution of all three ^99m^Tc-labeled constructs was compared at 24 h p.i. (Figure 8 and Table S4). At this time-point, the uptake of ^99m^Tc-ADAPT6-ABD-AA in blood was two-fold higher than the uptake of ^99m^Tc-ADAPT6-ABD-mcDM1. The uptake of ^99m^Tc-ADAPT_Neg_-ABD-mcDM1 in liver was significantly (*p* < 0.05) higher than ^99m^Tc-ADAPT6-ABD-mcDM1 and ^99m^Tc-ADAPT6-ABD-AA. Conversely, the renal uptake of ^99m^Tc-ADAPT_Neg_-ABD-mcDM1 was lower than the other two. The uptake in the tumor of ^99m^Tc-ADAPT6-ABD-mcDM1 and ^99m^Tc-ADAPT6-ABD-AA was significantly higher than the uptake of ^99m^Tc-ADAPT_Neg_-ABD-mcDM1, showing active targeting of the tumors by ADAPT6. The small uptake of ^99m^Tc-ADAPT_Neg_-ABD-mcDM1 detected in the tumor is likely a result of the enhanced permeability and retention (EPR) effect.

**Figure 9:**
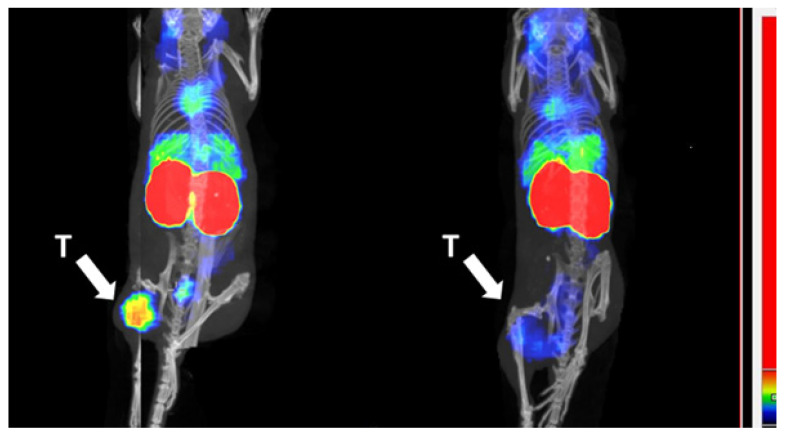
Imaging and tumor targeting specificity of ^99m^Tc-ADAPT6-ABD-mcDM1. SPECT/CT images (maximum intensity projections). The left image shows a mouse after injection of ADAPT6-ABD-mcDM1 and the right picture shows an animal where the tumor had been pre-saturated by injection of a large amount of trastuzumab. The arrow with the letter “T” points to the tumors, which were implanted in a hind leg. The color scale sidebar shows relative activity.

To visualize the biodistribution and to further demonstrate the HER2-dependent targeting by ^99m^Tc-ADAPT6-ABD-mcDM1, an in vivo blocking experiment was performed. Radiolabeled ADAPT6-ABD-mcDM1 was injected into mice with SKOV-3 xenografts, with or without pre-injection of an excess of trastuzumab to block available HER2 receptors, and SPECT/CT images were recorded at 24 h after injection (Figure 9). For the non-blocked mouse, a high uptake was observed in the tumor and in the kidneys. For the blocked mouse, a high uptake was observed in the kidneys, but the uptake in the tumor was appreciably lower.

## 4. Discussion

Engineered scaffold proteins (ESPs) capable of specific delivery of cytotoxic compounds to cancer cells have the potential to become agents for targeted cancer therapy in the future. However, different scaffold proteins have different structures and surface amino acids, which influence their distribution properties and off-target interactions. This necessitates a careful evaluation of every type of ESP for their suitability as targeting agent. The results of this study suggest that the relatively unexplored ADAPT-class of ESPs might be suitable for targeted delivery of anti-cancer drugs.

Recombinant expression and purification of ADAPT6-ABD, as well as conjugation of mcDM1, was straightforward. After successful purification and conjugation, the drug conjugate could specifically bind to, get internalized, and efficiently kill cells with high HER2 expression (Figure 3 and Figure 4). A difference in cytotoxic potency was noted among the high-HER2 expressing cell lines. ADAPT6-ABD-mcDM1 showed efficient killing of the breast cancer cell lines SKBR3 and AU565, but the ovarian cancer cell line SKOV-3 was considerably more resistant to DM1 treatment. Previous studies of ADCs have demonstrated different responses in different cell lines with a high HER2 expression level [36,37,38]. It was suggested that the differences might be related to differences in the internalization rate of the receptor, expression level of multi-drug resistance proteins, and differences in the efficiency of lysosomal degradation. In our previous studies with anti-HER2 affibody molecules, conjugated with drugs and toxins, the SKOV-3 cell line was also more resistant to targeted treatment in vitro than other cell lines with a similarly high HER2 expression level [11,39].

ADAPT6-ABD-mcDM1, the non-toxic control ADAPT6-ABD-AA, and the non-target control ADAPT_Neg_-ABD-mcDM1 could be stably radiolabeled with ^99m^Tc (Table S2). The ADAPT6-containing constructs showed high-affinity binding to living cells with high HER2 expression when analyzed with a LigandTracer biosensor, however the binding was characterized by two interactions, one with stronger and one with weaker affinity (Figure 6). The association rates of both interactions were similar, but the dissociation rates differed. Such types of multiple interactions could be due to the fact that HER2 is present on the membranes of living cells in different states; as a monomer or in a homo- or heterodimeric form. Monomers and dimers may have slightly different conformations, which in turn may affect the interaction. Previous studies have also reported this phenomenon for different binders to HER2 [31,40,41] and other HER family receptors [42]. The equilibrium dissociation constant for the strong interaction with SKOV-3 cells in the LigandTracer instrument was similar to the equilibrium dissociation constant for the interaction with the extracellular domain of HER2 in the Biacore experiment (Table 1).

The internalization rate of ^99m^Tc-ADAPT6-ABD-mcDM1 and ^99m^Tc-ADAPT6-ABD-AA was 20–38% of the cell-associated radioactivity and data were similar or slightly lower compared to those previously reported for the similar, affibody-based, drug conjugate ^99m^Tc-Z_HER2:2891_-ABD-mcDM1 (30–40%) [11]. Since internalization is critical for drug action, therapy using ADAPT6 conjugate might have a similar efficacy compared to the affibody variant.

The radiolabeled ^99m^Tc-ADAPT6-ABD-mcDM1 drug conjugate was further evaluated in nude mice bearing SKOV-3 xenografts. The fusion with ABD had appreciably expanded the residence of the construct in circulation, with a blood concentration of 11 ± 2% ID/g at 4 h after injection. It was significantly longer compared to the blood concentration of non-ABD fused ADAPT6 (^99m^Tc-(HE)_3_-ADAPT6), which was only 0.31 ± 0.05% ID/g at the same time point in a previous study [21]. The targeting specificity of ^99m^Tc-ADAPT6-ABD-mcDM1 was confirmed in two ways, by saturation of binding sites on HER2 using trastuzumab, which appreciably lowered tumor uptake, and by comparison with the tumor uptake of the non-targeted control, ^99m^Tc-ADAPT_Neg_-ABD-mcDM1 (Figure 7), which was appreciably lower than the uptake of ^99m^Tc-ADAPT6-ABD-mcDM1. The results of these tests clearly demonstrated HER2-specific accumulation of ADAPT6-ABD-mcDM1 in the tumors. The biodistribution results showed that the uptake in the tumor increased up to 24 h p.i. where it reached a plateau of 5% ID/g. This was lower compared to the homologous ADAPT6-ABD labeled at the C-terminus with ^177^Lu, ^177^Lu-DOTA-ABD_035_-ADAPT6, which has been evaluated in a similar mouse model with SKOV-3 tumor xenografts [28]. In that study, the tumor uptake increased up to 26 ± 4% ID/g at 24 h. The higher tumor uptake of ^177^Lu-labeled construct is likely a consequence of its higher blood radioactivity, 17 ± 2% IA/g at 24 h, and thus higher bioavailability compared to ^99m^Tc-ADAPT6-ABD-mcDM1, with a blood radioactivity of 5.0% ID/g at 24 h. At 24 h, the uptake in liver of ^177^Lu-DOTA-ABD_035_-ADAPT6 (4.8 ± 0.3% ID/g) and technetium-99 labeled ADAPT6-ABD-mcDM1 (4.83 ± 0.27% ID/g) were similar, but the uptake in the kidneys was significantly lower for ^177^Lu-DOTA-ABD_035_-ADAPT6 (10.9 ± 0.7% ID/g) than for ADAPT6-ABD-mcDM1 (83 ± 4% ID/g). This strongly suggests a low and similar clearance rate of both constructs through the liver, but a more rapid clearance of ^99m^Tc-ADAPT6-ABD-mcDM1 through the kidneys. The Biacore experiments with ADAPT6-ABD-mcDM1 confirmed reasonably strong binding to serum albumins. However, DM1 still appears to influence the binding properties of ABD, since the blood clearance is faster and kidney uptake is higher compared to the ^177^Lu-labeled construct. It would be interesting to investigate this phenomenon further in future studies, and possibly to compare the ABD-technology with other methods of plasma half-life extension.

Another class of ESPs are the affibody molecules (58 amino acids), which are slightly larger than ADAPTs (46 amino acids), and similarly folded into three-helix bundle domains. An affibody molecule with strong and specific affinity for HER2 has previously been expressed as a fusion to ABD_035_ and derivatized with mcDM1 to Z_HER2_-ABD-mcDM1 [11], a conjugate analogous to ADAPT6-ABD-mcDM1. In the present study, the cytotoxic potential (IC_50_ value) of ADAPT6-ABD-mcDM1 were 5 nM, 7 nM, and 80 nM for SKBR3, AU565 and SKOV-3 cells, respectively. In a similarly performed experiment, the IC_50_ values for Z_HER2_-ABD-mcDM1 was 0.6 nM, 1 nM, and 33 nM towards the same cell lines. A partial explanation for the weaker cytotoxic potential of ADAPT6-ABD-mcDM is most likely its weaker affinity for HER2 (6 nM) compared to the affinity of Z_HER2_-ABD-mcDM1 for HER2 (0.7 nM) [11]. Further, the cytotoxic potential of T-DM1 towards the same cell lines has previously been determined to 0.2 nM, 0.2 nM, and 0.5 nM, respectively [10], which is stronger than ADAPT6-ABD-mcDM1, particularly for the SKOV3 cell line. In this case, the cytotoxic potential appears to be cell line dependent since the relative difference between ADAPT6-ABD-mcDM1 and T-DM1 is not the same for the three cell lines. It should also be noted that an antibody and an ADAPT are quite different in terms of biochemical behavior, and the linker connecting DM1 differs, which makes it difficult to draw conclusions concerning the reason for the difference in cytotoxic potential between the two.

The biodistribution of technetium-99 labeled Z_HER2_-ABD-mcDM1 showed a similar pattern to ^99m^Tc-ADAPT6-ABD-mcDM1, which was characterized by a low unspecific uptake in normal organs except for the kidneys. ^99m^Tc-Z_HER2_-ABD-mcDM1 had a slightly higher blood retention at 24 h (6.7 ± 0.3 versus 3.9 ± 0.5% ID/g), and a slightly longer plasma half-life of 12.5 h compared to 9.0 h for ^99m^Tc-ADAPT6-ABD-mcDM1. The uptake of both constructs in the tumor was similar, 6.7 ± 0.3% ID/g for ^99m^Tc-Z_HER2_-ABD-mcDM1 compared to 5.5 ± 1.8% ID/g for ^99m^Tc-ADAPT6-ABD-mcDM1. Interestingly, ^99m^Tc-Z_HER2_-ABD-mcDM1 also had high renal uptake. However, it could successfully be used for experimental therapy of SKOV-3 xenografts in mice, and a pathology investigation did not reveal any renal damage. Most likely, the explanation can be attributed to the mode of action of DM1, which is inhibition of tubulin polymerization and it should thus be more toxic for rapidly dividing cells. Apparently, the proliferation rate of proximal tubuli cells in the kidneys is slower, which permits accumulation of an appreciable amount of DM1 without any toxic effect. The same effect could, hopefully, be expected for ADAPT6-ABD-mcDM1.

In conclusion, we have shown that the relatively unexplored class of engineered scaffold proteins, the ADAPTs, can be fused with an ABD for extension of residence in circulation and site-specifically conjugated with the highly cytotoxic microtubulin inhibitor DM1. The resulting drug conjugate retained high affinity to HER2 and albumin. ADAPT6-ABD-mcDM1 was highly potent towards cells with high HER2 expression in vitro. ^99m^Tc-ADAPT6-ABD-mcDM1 accumulated specifically in HER2-expressing human xenografts in vivo. Its biodistribution in mice was characterized by low uptake in normal organs, except the kidneys. Taken together, the results show that ADAPTs are potentially suitable carriers of cytotoxic drugs to malignant tumors.

## Figures and Tables

**Table 1:** Kinetic parameters and equilibrium dissociation constants.

Analyte	Ligand	k_a_ (M^−1^·s^−1^)	k_d_ (s^−1^)	K_D_ (M)
ADAPT6-ABD-mcDM1	HER2	5.52 × 10^4^	3.12 × 10^−4^	5.65 × 10^−9^
	HSA	1.08 × 10^5^	5.89 × 10^−5^	5.43 × 10^−10^
	MSA	2.11 × 10^5^	4.42 × 10^−3^	2.09 × 10^−8^
ADAPT6-ABD-AA	HER2	1.16 × 10^5^	4.00 × 10^−4^	3.44 × 10^−9^
	HSA	5.47 × 10^5^	5.22 × 10^−5^	9.54 × 10^−11^
	MSA	1.03 × 10^6^	4.02 × 10^−3^	3.90 × 10^−9^
ADAPT_Neg_-ABD-mcDM1	HER2	ND ^a^	ND	ND
	HSA	4.11 × 10^5^	6.80 × 10^−5^	1.65 × 10^−10^
	MSA	7.90 × 10^5^	5.18 × 10^−3^	6.55 × 10^−9^

^a^ Not determined.

## Data Availability

Data are contained within the article.

## Supplementary Materials

The following are available online at https://www.mdpi.com/article/10.3390/pharmaceutics13111847/s1. Figure S1: SDS-PAGE analysis; Figure S2: Analysis of HER2 expression; Figure S3:Analytical RP-HPLC analysis of technetium-99 labeled constructs; Table S1: Molecular weights of the constructs; Table S2: radiochemical yields of radiolabeled ADAPT constructs; Table S3: Biodistribution of ^99m^Tc-ADAPT6-ABD-mcDM1 in BALB/c nu/nu mice bearing SKOV-3 xenografts at 1, 4, 24, and 48 h after injection; Table S4: biodistribution of technetium-99 labeled constructs in BALB/c nu/nu mice bearing SKOV-3 xenografts at 24 h after injection.
