# Supplementary material for: Targeting HER2 Expressing Tumors with a Potent Drug Conjugate Based on an Albumin Binding Domain-Derived Affinity Protein
Source: Pharmaceutics. 2021 Nov 3;13(11):1847. doi: 10.3390/pharmaceutics13111847 (PMC8619933; doi:10.3390/pharmaceutics13111847)
Supplement: Supplementary file 1 [file pharmaceutics-13-01847-s001.zip › pharmaceutics-1396686-supplementary.pdf]

pharmaceutics

MDPI

Supplementary Materials: Targeting HER2 Expressing Tumors with a Potent Drug Conjugate Based on an Albumin Binding Domain-Derived Affinity Protein

Javad Garousi, Haozhong Ding, Emma von Witting, Tianqi Xu, Anzhelika Vorobyeva, Maryam Oroujeni, Anna Orlova, Sophia Hober, Torbjörn Gräslund and Vladimir Tolmachev

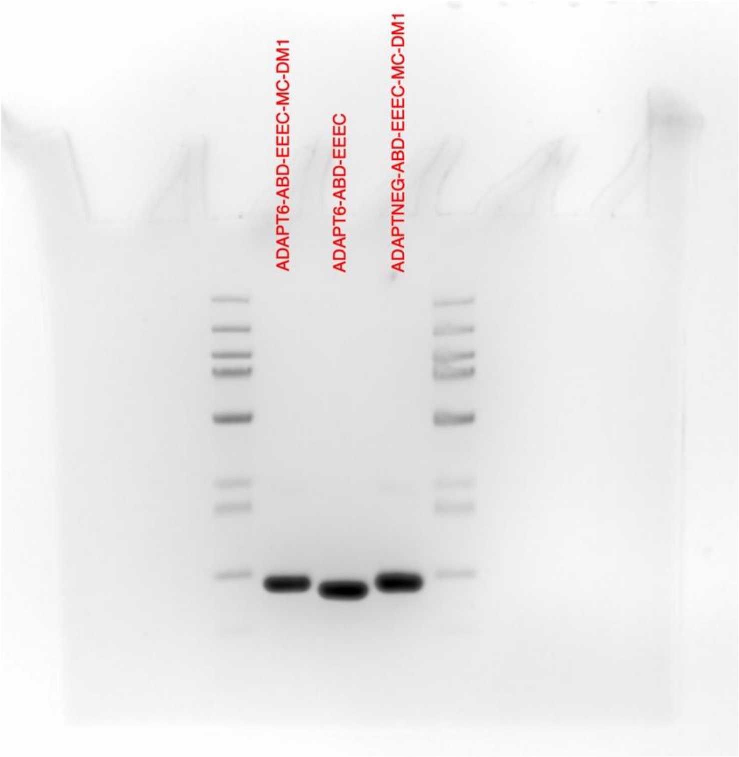

Figure S1. SDS-PAGE analysis. Separation of ADAPT6-ABD-mcDM1 (lane 2 from the left), ADAPT6-ABD-AA (lane 3 from the left) and $ADAPT_{Neg}$-ABD-mcDM1 (lane 4 from the left) by SDS-PAGE under reducing conditions. 5 µg material was loaded in each lane. Marker proteins were loaded in lane 1 and 5. The same gel is partially displayed in Figure 1B.

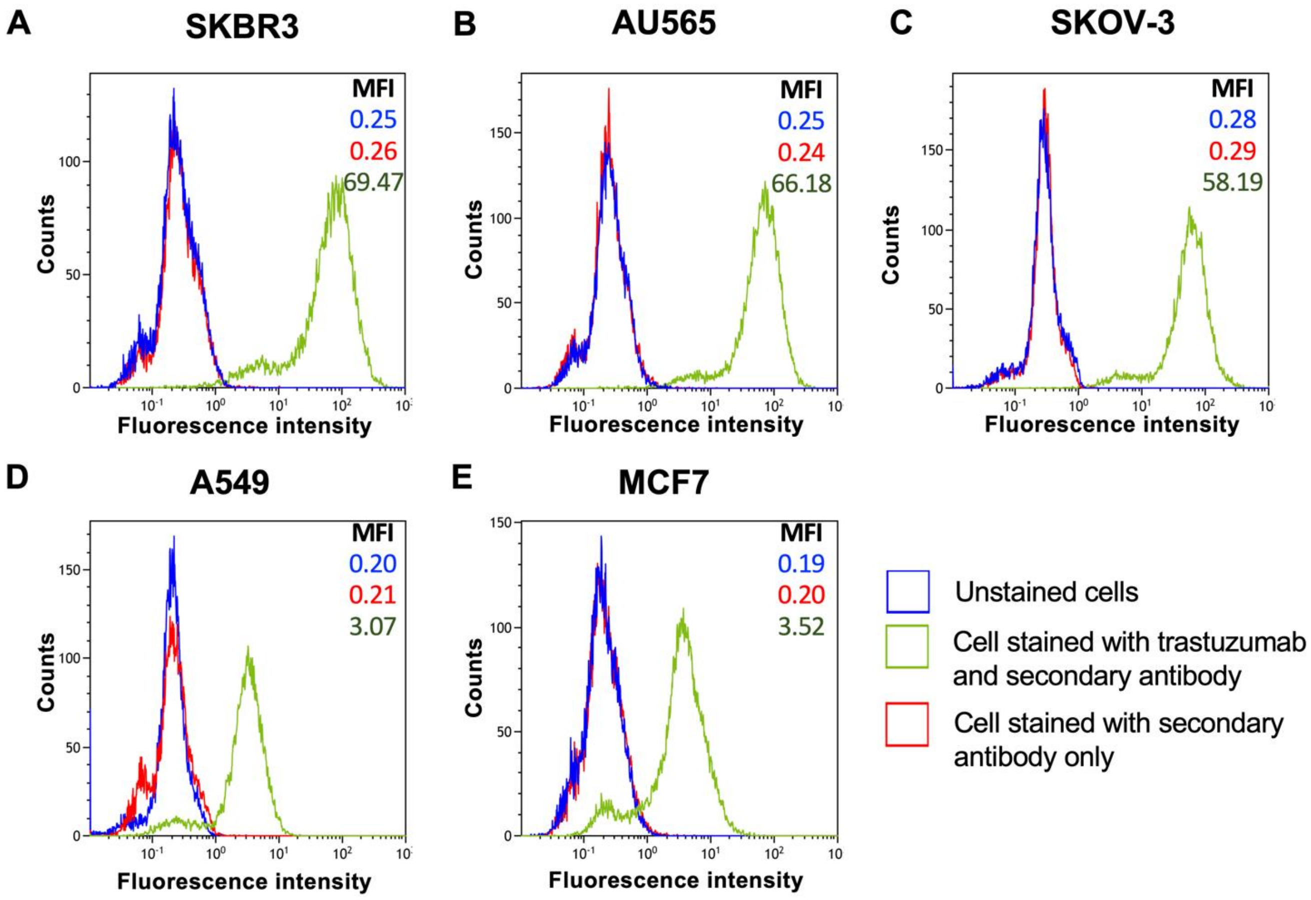

Figure S2. Analysis of HER2 expression. For each sample, 200,000 cells were stained with 5 µg/mL trastuzumab (Roche, Basel, Switzerland) for 15 min at room temperature, and washed twice with PBS with 3% BSA. After that, the cells were stained with the secondary Goat anti-Human IgG (H+L) Alexa 647 conjugate (Invitrogen, Waltham, Massachusetts, United States) for 15 min at room temperature followed by washing with PBS with 3% BSA. The samples were analysed by flow cytometry on a Gallios flow cytometer (Beckman Coulter, Brea, CA, United States) and 20,000 events were recorded for each sample. The cell line is indicated above each panel and the MFI in each panel corresponds to the geometric mean of the major peak for each curve.

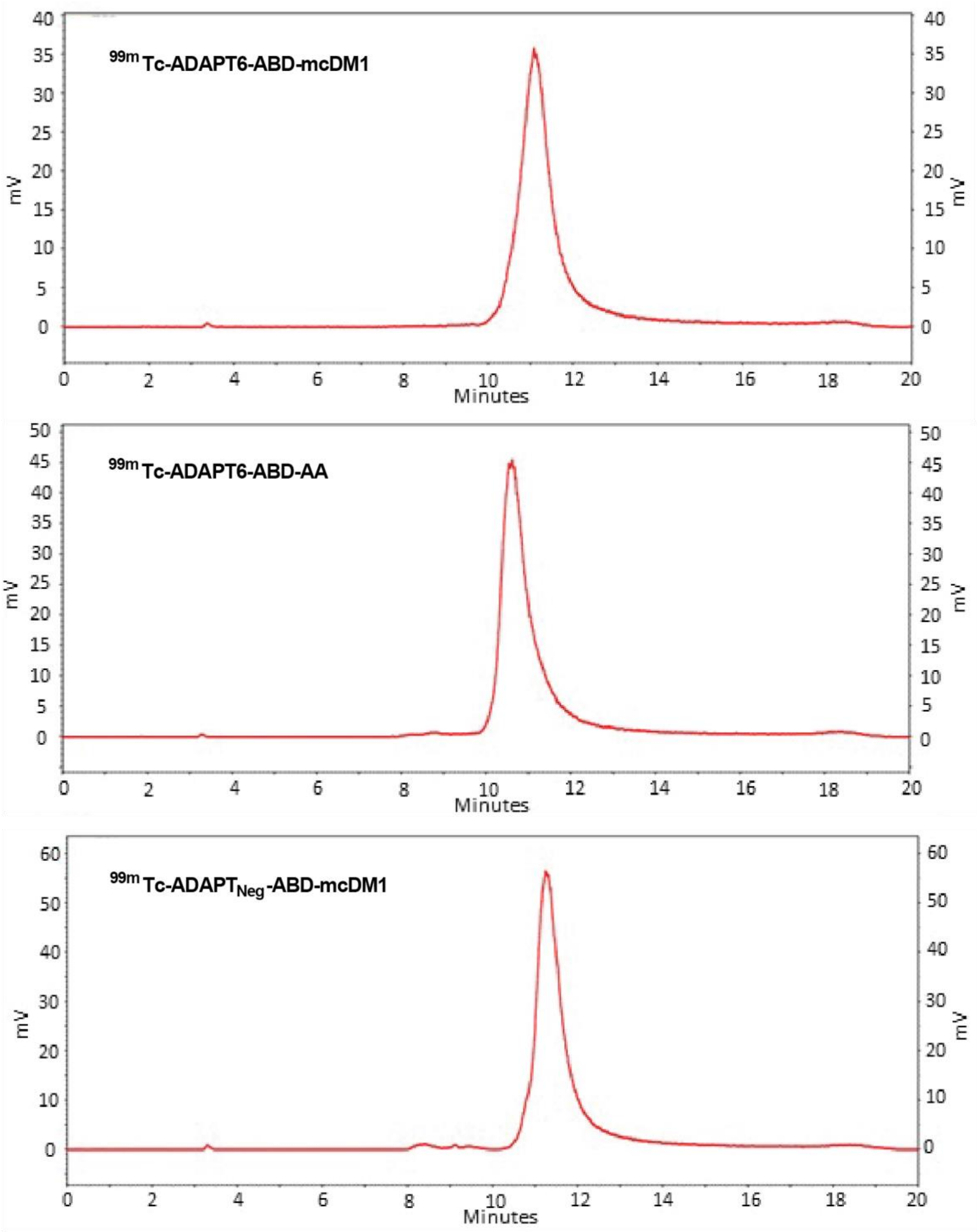

Figure S3. Analytical RP-HPLC analysis of technetium-99 labeled constructs. The panels show radio RP-HPLC chromatograms of ^{99m}Tc-ADAPT6-ABD-mcDM1, ^{99m}Tc-ADAPT6-ABD-AA and ^{99m}Tc-ADAPT$_{Neg}$-ABD-mcDM1. All three constucts are eluted as single peaks showing homogenously radiolabeled constructs.

Table S1. Molecular weights of the constructs.

Construct.	Calculated MW (Da)	Found [a] MW (Da)
ADAPT$_6$-ABD-mcDM1	13,722.9	13,722.0
ADAPT$_6$-ABD-AA	12,936.5	12,935.7
ADAPT$_{neg}$-ABD-mcDM1	13,606.7	13,605.9

[a] The molecular weights were measured by LC-MS.

Table S2. Radiochemical yields of radiolabeled ADAPT constructs.

ADAPT6 constructs	Radiochemical yield (%)	Radiochemical purity (%)	Radiochemical purity after histidine challenge (%) [a]
^{99m}Tc-ADAPT6-ABD-cmDM1	82±13	>99	>99
^{99m}Tc-ADAPT6-ABD-AA	78±18	>99	>99
^{99m}Tc-ADAPT$_{Neg}$-ABD-mcDM1	78±3	>99	>99

[a] After incubation with a 5000-fold molar excess of histidine.

Table S3. Biodistribution of ^{99m}Tc-ADAPT6-ABD-mcDM1 in BALB/c nu/nu mice bearing SKOV-3 xenografts at 1, 4, 24 and 48 h after injection. Data are expressed as %ID/g and are mean values from 4 animals ± 1 SD.

	1h	4h	24h	48h
Blood	15.93 ± 1.52 [a,b,c]	10.81 ± 1.76 [a,d,e]	3.93 ± 0.54 [b,d]	2.36 ± 0.09 [c,e]
Salivary glands	2.30 ± 0.50	2.21 ± 0.56 [e]	1.52 ± 0.25	1.20 ± 0.05 [e]
Lung	6.48 ± 0.73 [a,b,c]	4.65 ± 0.87 [a,e]	2.09 ± 0.25	1.45 ± 0.10 [c,e]
Liver	6.51 ± 0.33c	5.96 ± 1.07 [e]	4.83 ± 0.27	3.95 ± 0.14 [c,e]
Spleen	4.00 ± 0.39 [b,c]	3.03 ± 0.84	2.11 ± 0.23	1.83 ± 0.19 [c]
Small intestine	2.37 ± 0.40c	1.68 ± 0.45	0.84 ± 0.35	0.53 ± 0.08 [c]
Stomach	1.74 ± 0.54 [b,c]	1.19 ± 0.24 [e]	0.60 ± 0.06 [b,f]	0.39 ± 0.01 [c,e,f]
Large intestine	1.70 ± 0.39 [b,c]	1.40 ± 0.31 [e]	0.74 ± 0.12 [b,f]	0.54 ± 0.14 [c,e,f]
Kidney	198.95 ± 8.70 [b,c]	163.61 ± 14.70 [d,e]	82.72 ± 3.53 [b,d,f]	53.23 ± 3.93 [c,e,f]
Tumor	2.98 ± 1.12 [c]	5.11 ± 1.62	5.5 ± 1.80	5.20 ± 1.40 [c]
Muscle	0.9 ± 0.23 [c]	0.81 ± 0.05 [d,e]	0.51 ± 0.04 [d]	0.38 ± 0.05 [c,e]
Bone	1.78 ± 0.24 [b,c]	1.53 ± 0.43	0.94 ± 0.09 [b,f]	0.75 ± 0.14 [c,f]
GI tract	3.06 ± 0.64 [b,c]	2.69 ± 0.75 [e]	1.54 ± 0.33 [b]	0.91 ± 0.04 [c,e]

[a] Significant difference ($p < 0.05$) between 1 and 4 h; [b] Significant difference ($p < 0.05$) between 1 and 24 h.
[c] Significant difference ($p < 0.05$) between 1 and 48 h; [d] Significant difference ($p < 0.05$) between 4 and 24 h.
[e] Significant difference ($p < 0.05$) between 4 and 48 h; [f] Significant difference ($p < 0.05$) between 24 and 48 h.

Table S4. Biodistribution of technetium-99 labeled constructs in BALB/c nu/nu mice bearing SKOV-3 xenografts at 24 h after injection. Data are expressed as % ID/g and are mean values from 4 animals ± 1 SD.

	^{99m}Tc-ADAPT6-ABD-mcDM1	^{99m}Tc-ADAPT6-ABD-AA	^{99m}Tc-ADAPT$_{Neg}$-ABD-mcDM1	^{99m}Tc-ADAPT6-ABD-mcDM1 (block) [g]
Blood	3.93 ± 0.54 [a,b]	8.38 ± 0.66 [a,e]	8.76 ± 0.58 [b,f]	4.84 ± 0.35 [e,f]
Salivary glands	1.52 ± 0.25 [a,b]	2.53 ± 0.49 [a,e]	2.51 ± 0.12 [b,f]	1.65±0.10 [e,f]
Lung	2.09 ± 0.25 [a,b]	4.02 ± 0.33 [a,e]	4.26 ± 0.05 [b,f]	2.65±0.27 [c,f]
Liver	4.83 ± 0.27 [b]	4.24 ± 0.23 [d]	5.76 ± 0.45 [b,d,f]	4.76±0.14 [f]
Spleen	2.11 ± 0.23 [b]	2.98 ± 0.23	3.64 ± 0.67 [b,f]	2.58±0.33 [f]
Small int.	0.84 ± 0.35	1.36 ± 0.40	1.35 ± 0.25	0.88±0.14
Stomach	0.60 ± 0.06 [a,b]	0.93 ± 0.08 [a,e]	1.00 ± 0.08 [f]	0.72±0.05 [e,f]
Large int.	0.74 ± 0.12 [a,b]	1.03 ± 0.11 [a]	1.16 ± 0.12 [b,f]	0.85±0.06 [f]
Kidney	82.72 ± 3.53 [b]	87.75 ± 8.28 [d]	57.79 ± 1.41 [b,d,f]	88.44±8.66 [f]
Tumor	5.50 ± 1.80 [b,c]	7.52 ± 0.99 [d,e]	2.60 ± 0.28 [b,d]	2.02±0.32 [c,e]
Muscle	0.51 ± 0.04 [a,b]	0.78 ± 0.09 [a]	0.89 ± 0.08 [b,f]	0.60±0.12 [f]
Bone	0.94 ± 0.09 [a,b]	1.29 ± 0.07 [a]	1.47 ± 0.22 [b,f]	1.03±0.08 [f]
GI tract	1.54 ± 0.33	1.91 ± 0.23	2.12 ± 0.31	1.53±0.27

[a] Significant difference ($p < 0.05$) between the uptake of ^{99m}Tc-ADAPT6-ABD-mcDM1 and ^{99m}Tc-ADAPT6-ABD-AA.
[b] Significant difference ($p < 0.05$) between the uptake of ^{99m}Tc-ADAPT6-ABD-mcDM1 and ^{99m}Tc-ADAPT$_{Neg}$-ABD-mcDM1
[c] Significant difference ($p < 0.05$) between the uptake of ^{99m}Tc-ADAPT6-ABD-mcDM1 and ^{99m}Tc-ADAPT6-ABD-mcDM1 (block).
[d] Significant difference ($p < 0.05$) between the uptake of ^{99m}Tc-ADAPT6-ABD-AA and ^{99m}Tc-ADAPT$_{Neg}$-ABD-mcDM1.
[e] Significant difference ($p < 0.05$) between the uptake of ^{99m}Tc-ADAPT6-ABD-AA and ^{99m}Tc-ADAPT6-ABD-mcDM1 (block).
[f] Significant difference ($p < 0.05$) between the uptake of ^{99m}Tc-ADAPT$_{Neg}$-ABD-mcDM1 and ^{99m}Tc-ADAPT6-ABD-mcDM1 (block).
[g] Available HER2 receptors in the animals were blocked by a preinjection of trastuzumab.
